# Clinical neurology during the COVID-19 pandemic – physicians in training perspective series

**DOI:** 10.25122/jml-2022-1001

**Published:** 2022-01

**Authors:** Irina Benedek, Elian Hapca, Vitalie Vacaras, Dafin Mureșanu

**Affiliations:** 1. Department of Neuroscience, Iuliu Hatieganu University of Medicine and Pharmacy, Cluj-Napoca, Romania; 2. RoNeuro Institute for Neurological Research and Diagnostic, Cluj-Napoca, Romania

## Introduction

The COVID-19 pandemic has profoundly impacted access to medical care throughout the world. Due to the rapid spread of the SARS-CoV-2 worldwide, swift reorganization of the health care services had to be implemented to control the epidemiological context. Lockdowns have disproportionately negatively impacted vulnerable patients (*i.e.*, with several risk factors such as advanced age and different degrees of disability), generally leading to poorer outcomes [1]. Healthcare workers have been increasingly exposed to the virus, and patients postponed or canceled their medical visits due to fear of being infected, as health systems focused predominantly on COVID-19, neglecting other patients’ needs. As a consequence of physical distancing, hospital beds capacity was reduced, training and educational activities of physicians were limited or postponed, research activity was severely disrupted, and work conditions became more and more difficult. These indirect implications of COVID-19 were initially overshadowed by general uncertainty and race to understand the new threat.

Neurology and other specialties alike have adapted during these new times. For example, replacing the much-needed face-to-face medical visits with telemedicine for clinical practice has challenged the classic evaluation process, as diagnostic tools are limited in virtual settings. In addition, work-life balance, psychological wellbeing, work conditions, new treatment protocols, barriers to education, ethical implications, and many other issues interfered with optimal patient care pathways, eliciting general frustration and dissatisfaction.

In this editorial, we discuss quintessential experiences encountered by clinicians during clinical practice in the context of the COVID-19 pandemic, as reported by neurologists in training from the County Emergency Hospital in Cluj-Napoca, Romania.

## Neurological disease management in pandemic times

Acute stroke remains a major neurological emergency; therefore, quick establishments of safe and time-effective guidelines are needed. Unfortunately, despite the technical success, which did not suffer significant changes compared to the pre-pandemic years, a significant increase in the time between hospital admissions was registered. Potential causes of this lag include anxiety related to possible infection, discouraged patients from presenting to the hospital, ambulances and critical care units overload due to numerous COVID-19 emergencies and redirection of a considerable number of patients from non-COVID hospitals. Since the protection of healthcare professionals was prioritized, stroke patients also underwent specific testing and a chest CT scan to determine whether they could be a possible source of contamination in the hospital. In this way, the risks to other patients and staff were reduced without significantly lengthening the “door-to-groin” time [2].

In addition to major neurological emergencies, the follow-up of patients with chronic conditions and the proper continuation of treatment provision has led to the use of electronic means previously considered rarely [1]. The concept of telemedicine was implemented back in 1950 when different hospital systems shared images and medical information by telephone. It started from exchanging radiographic images to connecting the patient with the physician in real-time. Telemedicine can offer a wide range of advantages for the patient. Examples include reducing overall expenses, spending less time away from family or work, and most importantly, increasing privacy and limiting exposure and risk of infection. Conversely, telemedicine has numerous concerns, such as low or uncertain diagnostic accuracy, poor reimbursement mechanisms, impact on the patient-neurologist relationship, data protection, computer literacy, and other technical barriers [3].

Another negative impact of the novel coronavirus is burnout syndrome experienced by healthcare professionals, including neurologists. The definition of “burnout” according to the International Classification of Diseases – 11 is “a syndrome resulting from chronic workplace stress that has not been successfully managed”. Inevitably, the most important repercussion of exhaustion is reduced professional efficiency. As a result of unprecedented demand on the healthcare system worldwide, the working schedule was also affected during the pandemic. To avoid spreading the infection, the medical staff was demanded to work in shifts that increased the working hours and the workload itself, leading to sleep deprivation and exposure to risks, particularly in settings where access to protective equipment is scarce. It was reported that physicians, especially residents, exhibited higher levels of psychological distress and even developed post-traumatic stress disorders while working with patients during the viral outbreak [4].

## Impact of COVID-19 on graduate medical training, research activities, and healthcare ethics

Sometimes, subtle patient features can be the clue to solving complex clinical cases. For example, such signs and symptoms often lead to various neurological diseases or guide the physician to the correct diagnosis. Moreover, clinical examination in the outpatient setting favors face-to-face interaction with the patient and offers the leverage for improving clinical skills for the physician in training, contrary to the necessary emerging use of telemedicine in neurology. Therefore, physicians’ training access to such bedside interaction is crucial for their development as specialists.

The COVID-19 pandemic has unequivocally altered the process of achieving and spreading quality medical knowledge and has limited the amount of time available to interact with attending physicians or experienced professors in favor of virtual courses and conferences. The dissatisfaction of young neurologists reflects this in training and limited exposure to the vast clinical practice their supervisor could pass on to the young generation.

Moreover, medical research activity was also subjected to adjustments to comply fully with the epidemiological situation requirements. For example, some clinical trials involving in-person visits had to be suspended, postponed, or COVID-19 regulations had to be made to ensure both subjects and research staff were safe, aspects that led to an additional financial and psychological burden. Also, fellowships and grants were suspended or postponed, and career plans have taken an unexpected turnover.

The aspects mentioned above were outlined in several studies that focused on the quality of neurology training programs in this centennial epidemiological situation. The Resident and Research Fellow Section of the European Academy of Neurology gathered important data from the members regarding satisfaction of the training programs and difficulties encountered during this process in the current situation. It was noted that 53% of the respondents who had classes/educational activities were deprived of participating due to temporary suspension. Time spent with the patient or supervision of their work suffered a vital reduction. 79% of the responders claimed this pandemic would impact their training and career.

Regarding research activities during their residency program, 56% of them were altered due to pandemics. In the Ph.D. students/research fellows’ group, there was a 62% temporary suspension of research projects, 20% had to suspend or postpone their planned fellowship, and 17% of them stopped their fellowship. In addition, 56% of the residents were summoned to work in COVID-19 units due to the epidemiological situation, and 58% stated they were not ready to manage critical patients [5].

The Italian Society of Neurology conducted a similar study on all neurology residents attending 36 teaching hospitals in Italy during May 2020:

• Almost 30% of the respondents were redistributed to COVID-19 units;
• Residents used telemedicine to substitute about one-third of emergencies and outpatient interactions;
• Research activities were interrupted or reduced in 59% of cases and transferred to remote working if possible;
• An increase in work shifts and the number of residents recruited in COVID-19-dedicated units showed a North to South gradient following the epidemiology of the pandemic in Italy;
• Lessons and seminars were rescheduled online [6].

It is mandatory to acknowledge that the COVID-19 pandemic has had significant implications for neurologists in training, with the not-so-clear long-term impact. Given the current situation, teaching units with residents must ensure that their educational needs are satisfied in the best possible manner. Furthermore, as this epidemiological context persists, it is essential to optimize the training process by permanently collaborating and communicating with the residents, responding to their needs to avoid sacrificing the careers of the new generation of young neurologists.

The public health crisis has forced health care workers and neurologists to quickly adapt to the necessity of ensuring public welfare in contrast to individual patient care. Healthcare resources had to be distributed to save as many lives as possible under challenging circumstances. In this scenario, the care of the neurological patient, similar to a patient suffering from other diseases, is subject to many ethical conundrums such as:

• Need for fair access to medical care in the context of relocation of many hospital beds for COVID-19 patients and limited outpatient face-to-face visits;
• Dependence on others for personal needs of patients suffering from neurodegenerative and neuropsychiatric disorders;
• Need for psychological support in palliative care in many neurological diseases, such as neurodegenerative diseases or stroke;
• Respect for particular wishes of patients regarding medical treatment or personal values by the physicians and surrogate decision-makers;
• End-of-life interactions with patient families.

For medical specialists, the Hippocratic Oath must be respected even in critical times or complex scenarios, including end-of-life medical decisions and the limiting of undesired invasive treatments. Physicians have the ethical duty to provide equitable patient care, minimize harm, respect autonomy, and maintain trust, although they are faced with multiple moral dilemmas [7]. In terms of disease management and access to care, neurologists should make their patients and surrogate decision-makers aware of the pandemic’s effect on inpatient and outpatient care for the best risk-benefit assessment. However, because of limited face-to-face interactions with the patients, proper discussions about the neurologic illness, including the presence of a surrogate decision-maker to act as the patient’s voice, are restricted. In the case of end-of-life discussions, the impossibility of family members to be present at the bedside produces frustration and complicated grief. Also, surrogate decision-makers may choose to continue aggressive care because of poor understanding of the severity of the disease or guilt that results from the inability to visit. All these situations may unintentionally harm patients and families [8]. Considering these challenging times for clinical neurology, the American Academy of Neurology published ethical guidance on patient care during the pandemic, aiming “*to offer an overview and guidance on the ethical dimensions of care of patients with neurologic conditions during the COVID-19 pandemic”*. It is meant to be used by leaders and individual clinicians who find themselves adapting to the crisis. It also has the role of “*assisting in the development of regional scarce resource allocation policies and assisting triage committees in making the difficult decisions they may confront”* [9].

Such initiatives must be ubiquitous, focusing the efforts of every health care system in providing the best care for the patient, the safety of health care workers, the efficient allocation of resources, and many other ethical aspects that arise in the context of a pandemic. Permanent self-improvement is imperative, and lessons must be learned to strengthen knowledge on this topic because this epidemiological situation can escalate or repeat anytime.

## Conclusions and recommendations for the future

The COVID-19 crisis launched many difficulties in medical practice, particularly in the neurological field. Physicians had to adapt to the new situations quickly, learning to provide health services through telemedicine, facing reforms in patients’ care, high levels of psychological stress, burnout, working in COVID-19 departments out of their comfort zone, fear of being infected or infecting their loved ones, sacrificing their training, being exposed to new ethical dilemmas and many other unexpected aspects.

Although this particular context challenges the delivery of medical services, we must reflect on the weaker points of health systems, engage in better collaboration with key institutions, and learn the critical lesson that authorities must protect, organize and ensure proper health financing. Finally, we highlight the necessity to increase physicians’ professional satisfaction to provide the best health care in these tough times.
